# Strengthening Advance Care Planning and DNACPR Documentation in an Older Adult Psychiatric Inpatient Setting: A Quality Improvement Initiative

**DOI:** 10.1192/bjo.2026.11458

**Published:** 2026-06-30

**Authors:** Omolara Ogunleye, Omar Mahfoudi, Zobya Khan, Jeeano Paul, Sukran Altun

**Affiliations:** NELFT, London, United Kingdom

## Abstract

**Aims:**

Older adult psychiatric patients frequently present with significant physical comorbidities and frailty when admitted. Despite national DNACPR guidance emphasising proactive, individualised resuscitation planning and shared decision-making, advance care planning is often given lower priority in mental health inpatient settings. The Mental Capacity Act (2005) mandates structured capacity assessment and best interests decision-making where appropriate, yet documentation of resuscitation status can remain unclear orabsent. Baseline review on a 10-bed older adult male psychiatric ward demonstrated that 0% of patients had documented DNACPR status or recorded evidence of resuscitation discussions, representing potential clinical, ethical and governance risk. This quality improvement project aimed to achieve 100% documentation of DNACPR status and increase documented patient and/or family discussions within a two-month period (December 2025–January 2026).

**Methods:**

Baseline measurement confirmed absence of DNACPR documentation or recorded discussions across all inpatients. Data were obtained retrospectively through electronic clinical record (RiO) review. Two sequential PDSA cycles were implemented. Cycle 1 embedded mandatory DNACPR status review into weekly multidisciplinary ward rounds, with documentation recorded in the electronic clinical record for all patients. Cycle 2 introduced clinician-led discussions with patients and/or families regarding resuscitation preferences; where patients lacked capacity, decisions were guided by Mental Capacity Act principles, including documented capacity assessment and best interests reasoning, and formal DNACPR documentation was completed where clinically appropriate. Outcome measures included: (1) percentage of patients with documented DNACPR status; (2) percentage with documented patient or family discussion; and (3) percentage with an active DNACPR form in place. Sustainability was supported by incorporating DNACPR review into the ward round documentation to embed the intervention within routine clinical review.

**Results:**

Following Cycle 1, documented DNACPR status increased from 0% to 100% of inpatients. Following Cycle 2, 50% of patients had documented resuscitation discussions and 30% had an active DNACPR order in place (baseline 0%). Discussions clarified previously undocumented patient preferences and facilitated structured best interests decision-making inpatients lacking capacity. Documentation compliance remained at 100% at four-week follow-up after completion of the intervention period, demonstrating sustained change beyond the initial implementation phase.

**Conclusion:**

Embedding DNACPR review within routine weekly ward processes resulted in rapid and sustained improvement in documentation and initiation of advance care planning in an older adult psychiatric setting. The intervention strengthened alignment with national guidance, improved preparedness for medical deterioration, and enhanced governance standards within the inpatient service. Although limited by small sample size and short follow-up duration, the project demonstrates a low-cost, scalable model for improving parity between physical and mental healthcare. We plan to extend this approach to a 20-bed female older adult ward on the same site and a 20-bed mixed older adult ward at another Trust site to promote consistent advance care planning practices across services.

